# Role of Mitochondrial Electron Transport Chain Complexes in Capsaicin Mediated Oxidative Stress Leading to Apoptosis in Pancreatic Cancer Cells

**DOI:** 10.1371/journal.pone.0020151

**Published:** 2011-05-25

**Authors:** Kartick C. Pramanik, Srinivas Reddy Boreddy, Sanjay K. Srivastava

**Affiliations:** Department of Biomedical Sciences and Cancer Biology Center, School of Pharmacy, Texas Tech University of Health Sciences Center, Amarillo, Texas, United States of America; Texas A&M University, United States of America

## Abstract

We evaluated the mechanism of capsaicin-mediated ROS generation in pancreatic cancer cells. The generation of ROS was about 4–6 fold more as compared to control and as early as 1 h after capsaicin treatment in BxPC-3 and AsPC-1 cells but not in normal HPDE-6 cells. The generation of ROS was inhibited by catalase and EUK-134. To delineate the mechanism of ROS generation, enzymatic activities of mitochondrial complex-I and complex-III were determined in the pure mitochondria. Our results shows that capsaicin inhibits about 2.5–9% and 5–20% of complex-I activity and 8–75% of complex-III activity in BxPC-3 and AsPC-1 cells respectively, which was attenuable by SOD, catalase and EUK-134. On the other hand, capsaicin treatment failed to inhibit complex-I or complex-III activities in normal HPDE-6 cells. The ATP levels were drastically suppressed by capsaicin treatment in both BxPC-3 and AsPC-1 cells and attenuated by catalase or EUK-134. Oxidation of mitochondria-specific cardiolipin was substantially higher in capsaicin treated cells. BxPC-3 derived ρ^0^ cells, which lack mitochondrial DNA, were completely resistant to capsaicin mediated ROS generation and apoptosis. Our results reveal that the release of cytochrome c and cleavage of both caspase-9 and caspase-3 due to disruption of mitochondrial membrane potential were significantly blocked by catalase and EUK-134 in BxPC-3 cells. Our results further demonstrate that capsaicin treatment not only inhibit the enzymatic activity and expression of SOD, catalase and glutathione peroxidase but also reduce glutathione level. Over-expression of catalase by transient transfection protected the cells from capsaicin-mediated ROS generation and apoptosis. Furthermore, tumors from mice orally fed with 2.5 mg/kg capsaicin show decreased SOD activity and an increase in GSSG/GSH levels as compared to controls. Taken together, our results suggest the involvement of mitochondrial complex-I and III in capsaicin-mediated ROS generation and decrease in antioxidant levels resulting in severe mitochondrial damage leading to apoptosis in pancreatic cancer cells.

## Introduction

Pancreatic cancer is one of the most deadliest of all the solid malignancies in the United States [1]. Efforts have been directed towards the development of adjuvant and neoadjuvant therapies in an attempt to improve survival rate [1]. Pancreatic cancers generally respond poorly to conventional treatment modalities such as chemotherapy and radiation therapy [2]. Unfortunately, the toxicity and inherent resistance of chemotherapeutic agent such as 5-fluorouracil (5-FU) and gemcitabine in pancreatic cancer are still reasons for poor prognosis [3]. There is no consensus regarding optimal therapeutic agents in pancreatic cancer, therefore the development of novel approaches to prevent and treat pancreatic cancer is an important mission. Epidemiological studies continue to support the premise that diet rich in fruits, vegetables and some spices may be protective against various human malignancies including pancreatic cancer and that consumption of chili peppers may protect against gastrointestinal-related cancers [4], [5], [6], [7], [8], [9], [10].

**Figure 1:**
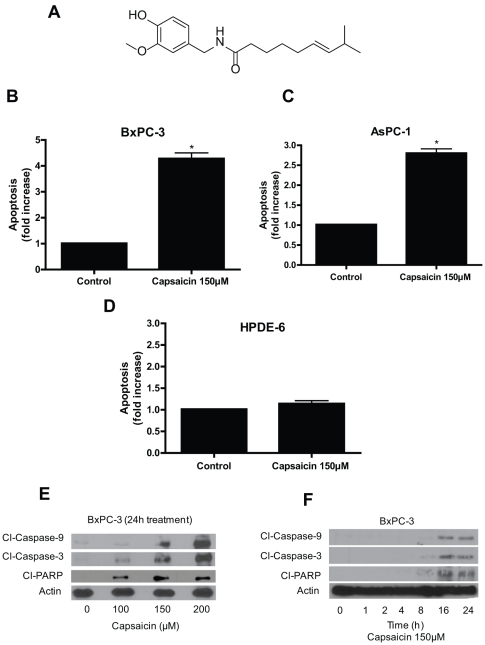
Capsaicin triggers apoptosis in pancreatic cancer cells but not in normal HPDE-6 cells. (*A*) Structure of capsaicin. Apoptosis inducing effects of capsaicin (150 µM, 24 h) in (*B*) BxPC-3, (*C*) AsPC-1 and (*D*) HPDE-6 cells, was determined using annexin-V/FITC and propidium iodide and analyzed by flow cytometery. Results are expressed as mean ± SD (n = 4) of four independent experiments. *Statistically different when compared with control as analyzed by student's t-test (*P<0.05*). (*E*) BxPC-3 cells were treated with different concentrations of capsaicin for 24 h and (*F*) Cell were treated at different time intervals with 150 µM capsaicin and analyzed by immunoblotting for cleavage of caspase-9, caspase-3 and PARP as described in Materials and Methods. Each blot was stripped and reprobed with anti-β-actin antibody to ensure equal protein loading. These experiments were performed three times independently with similar observation made in each experiment.

Capsaicin, a homovanillic acid derivative (N-vanillyl-8-methyl-nonenamide) is an active and spicy component of hot chili pepper (Fig. 1A) [11], [12] and has been used as food additive for long time throughout the world, particularly in South Asian and Latin-American countries [13], [14], [15], [16], [17]. This alkaloid has been used to treat pain and inflammation, associated with a variety of diseases [18], [19], [20], [21]. Several recent studies demonstrated that capsaicin has antiproliferative effect in hepatic [22] gastric [23] prostate [24] colon [25] and leukemic cells [26]. Capsaicin generally exerts its physiologic response by stimulating vanilloid 1 (TRPV-1) receptor, however, receptor independent effects of capsaicin have been observed in cancer cells [25], [26], [27]. Capsaicin suppresses the growth of cancer cells by NF-kB inactivation, ROS generations, cell-cycle arrest and modulating EGFR/HER-2 pathways [28], [29], [30], [31], [32], [33]. The exact molecular mechanism by which capsaicin causes oxidative stress and apoptosis remains vague. We have shown previously that capsaicin induced apoptosis in pancreatic cancer cells was associated with ROS generation and mitochondrial disruption [34]. However the exact mechanism by which capsaicin causes ROS generation and cell death was not clear.

In the present study, we demonstrate that capsaicin causes ROS (superoxide radical and hydrogen peroxide) generation by inhibiting mitochondrial complex-I and complex-III activity and ATP levels in BxPC-3 and AsPC-1 human pancreatic cancer cell lines, without affecting BxPC-3 derived ρ^0^ and normal HPDE-6 cells. At the same time catalase and glutathione peroxidase activity and expression were suppressed by capsaicin treatment. Supplementing the cells with PEG-catalase, PEG-SOD, EUK-134 (catalase mimick) or transfecting the cells with catalase almost completely blocked capsaicin mediated ROS generation and apoptosis. In addition, tumors from 2.5 mg/kg capsaicin treated mice exhibited decreased SOD activity and an increase in GSSG/GSH level. This study provides a direct evidence of how capsaicin utilizes mitochondria to cause oxidative stress leading to apoptosis in pancreatic cancer cells.

## Results

### Capsaicin triggers apoptosis in pancreatic cancer cells but not in normal HPDE-6 cells

Apoptosis was determined by flow cytometery using annexin-V/FITC and propidium iodide. Treatment of BxPC-3 and AsPC-1 cells with 150 µM capsaicin for 24 h resulted in about 2.5–5 folds increase in apoptosis (Fig. 1B–C). Interestingly, capsaicin failed to induce apoptosis in normal HPDE-6 cells (Fig. 1D). The apoptosis inducing effect of capsaicin was further confirmed by western blotting. As shown in Fig. 1E, capsaicin treatment caused significant activation of caspase-9, caspase-3 and PARP as evident by their respective cleavages in a concentration dependent manner. On the other hand, capsaicin treatment did not caused any cleavages of caspases or PARP in normal HPDE-6 cells (data not shown). In a time dependent study, cleavage of caspase 9/3 and PARP were evident by 16 and 24 h of capsaicin treatment (Fig. 1F).

### Capsaicin causes generation of mitochondrial ROS in pancreatic cancer cells

**Figure 2:**
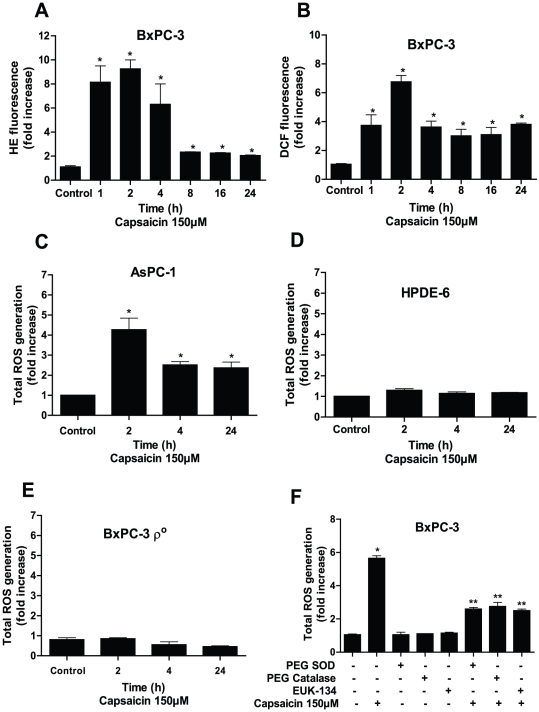
Capsaicin causes generation of mitochondrial ROS in pancreatic cancer cells. (*A*) and (*B*) BxPC-3 cells were treated with DMSO or 150 µM capsaicin for different time points and stained with HE and DCFDA and analyzed for superoxide radical and hydrogen peroxide respectively by flow cytometery. (*C*) AsPC-1, (*D*) HPDE-6, (*E*) BxPC-3 ρ^0^ cells were treated with 150 µM capsaicin for 2, 4 and 24 h and analyzed for total ROS generation (superoxide and hydrogen peroxide) by flow cytometer after staining the cells with HE and DCFDA. Results are expressed as mean ± SD (n = 3) from four independent experiments and data represents fold increase of ROS generation over control. *Statistically different when compared with control as analyzed by one-way ANOVA followed by Bonferroni's post-hoc test *P<0.05*). (*F*) Effect of antioxidants on capsaicin mediated total ROS generation in BxPC-3 cells. Cells were treated with PEG-SOD (100 U/ml), PEG-catalase (500 U/ml) or EUK-134 (50 µM) for 1 h followed by 150 µM capsaicin for 2 h. Results are expressed as mean ± SD (n = 3) of four independent experiments. *Statistically different compared with control (*P<0.05*) and **statistically different when compared with capsaicin treatment (*P<0.05*), as analyzed by one-way ANOVA followed by Bonferroni's post-hoc test.

Intracellular ROS generation by capsaicin was evaluated by flow cytometry using hydroethidine (HE) and DCFDA. As shown in Fig. 2A, in a time dependent study, capsaicin treatment caused about 8–9 folds increase in superoxide radical within 1–2 h which decreased by 24 h as measured by HE fluorescence by flow cytometery. Similarly the generation of hydrogen peroxide upon capsaicin treatment increased by 4–7 folds within 1–2 h and then decreased but maintained levels higher than superoxide by 24 h, as measured by DCF fluorescence by flow cytometery (Fig. 2B). The generation of ROS was as early as 1 h as compared with controls in BxPC-3 cells. In order to see whether antioxidants can block ROS generation, cells were pretreated with PEG-SOD (100 U/ml), PEG-catalase (500 U/ml) or 50 µM EUK −134 (a cell permeable catalase mimetic) prior to capsaicin treatment. PEG-SOD almost completely blocked superoxide radical generation whereas PEG-catalase completely blocked hydrogen peroxide generation as measured by HE and DCF fluorescence respectively by flow cyometery (Fig S1A–B). To confirm the specificity of antioxidants, we used PEG-catalase to block superoxide radical generation. As expected, PEG-catalase completely failed to block superoxide radical generation (Fig S1C). Similarly, PEG-SOD failed to block hydrogen peroxide generation (data not shown). In subsequent experiments, we measured total ROS (superoxide radical+hydrogen peroxide) generation. Similarly, capsaicin treatment increased total ROS generation by about 2.5–4.5 fold in AsPC-1 cells with maximum at 2 h of treatment (Fig. 2C). Capsaicin treatment did not cause any significant ROS generation in normal HPDE-6 cells, suggesting that normal cells are resistant to the effects of capsaicin (Fig. 2D). In a combination treatment, our results indicate that PEG-SOD, PEG-catalase and EUK-134 substantially blocked capsaicin mediated total ROS generation in BxPC-3 cells (Fig. 2F).

### BxPC-3 derived ρ^0^ cells were completely resistant to capsaicin mediated ROS generation

To firmly establish the contribution of mitochondria in ROS generation by capsaicin, we generated the ρ^0^ variants of BxPC-3 cells. ρ^0^ cells were generated and maintained by incubating BxPC-3 cells with 60 ng/ml ethidium bromide and 50 mg/ml of uridine for 12 weeks and characterized by PCR to confirm the depletion of mtDNA and normal oxidative phosphosrylation as reported previously [35]. The survival of ρ^0^ cells is dependent upon ATP derived from anaerobic glycolysis. ρ^0^ cells are unable to generate ROS from ETC complex as they lack normal oxidative phosphorylation [35], [36]. Compared to wild type BxPC-3 cells, total ROS generation was not at all observed in BxPC-3 ρ^0^ cells upon treatment with capsaicin (Fig. 2E). Taken together, our results suggest that BxPC-3 ρ^0^ cells were altogether resistant to the effects of capsaicin as compared with wild-type BxPC-3 cells.

### Capsaicin treatment inhibits ETC Complex-I and Complex-III activities

**Figure 3:**
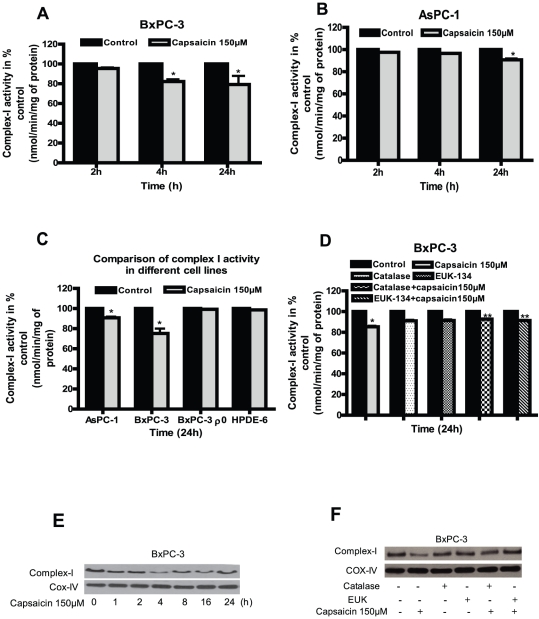
Involvement of ETC complex-I in capsaicin mediated ROS generation. Enzymatic activities of mitochondrial complex I was determined in the pure mitochondria isolated from control and 150 µM capsaicin treated (*A*) BxPC-3 and (*B*) AsPC-1 cells for 2, 4 and 24 h. (*C*) Comparison of complex-I activity in AsPC-1, BxPC-3, BxPC-3 ρ^0^ and HPDE-6 cells treated with 150 µM for 24 h. (*D*) Capsaicin mediated decrease of complex-I activity was prevented by pre-treatment of BxPC-3 cells with catalase (2000 U/ml) and EUK-134 (50 µM) for 1 h followed by 150 µM capsaicin for 24 h. Results are expressed over control as mean ± SD (n = 3) of three independent experiments. *Statistically different compared with control (*P<0.05*) and **statistically different when compared with capsaicin treatment (*P<0.05*), as analyzed by one-way ANOVA followed by Bonferroni's post-hoc test. (*E*) Complex-I protein expression was determined by immunoblotting using pure mitochondrial protein isolated from control and 150 µM capsaicin treated BxPC-3 cells for the indicated time periods or (*F*) 1 h pre treatment with catalase (2000 U/ml) or EUK-134 (50 µM) followed by 150 µM capsaicin for 24 h. Immunoblotting for each protein was performed three times independently and similar results were obtained. The blots were stripped and reprobed with anti-Cox-IV for mitochondrial proteins to ensure equal protein loading.

**Figure 4:**
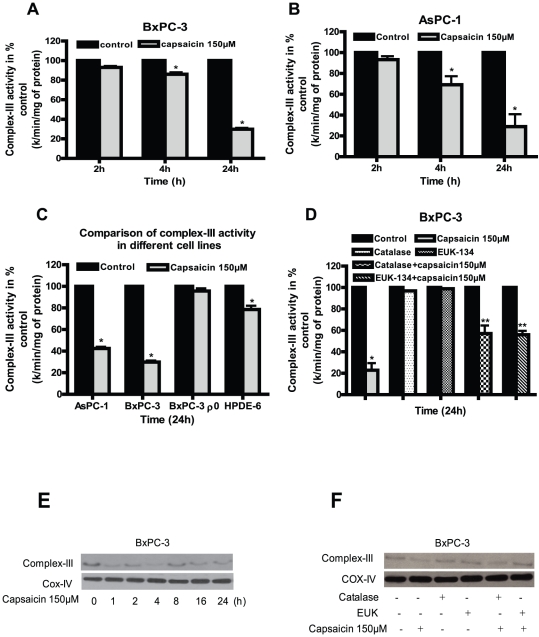
Involvement of ETC complex-III in capsaicin mediated ROS generation. Mitochondrial complex-III activity was determined in the pure mitochondria isolated from control and 150 µM capsaicin treated (*A*) BxPC-3 and (*B*) AsPC-1 cells for 2, 4 and 24 h. (*C*) Comparison of complex-III activity in AsPC-1, BxPC-3, BxPC-3 ρ^0^ and HPDE-6 cells treated with 150 µM capsaicin for 24 h. (*D*) Capsaicin mediated decrease of complex-III activity was attenuated by pre-treatment of BxPC-3 cells with catalase (2000 U/ml) or EUK-134 (50 µM) for 1 h followed by 150 µM capsaicin for 24 h. Results are expressed over control as mean ± SD (n = 3) of four independent experiments. *Statistically different compared with control (*P<0.05*) and **statistically different when compared with capsaicin treatment (*P<0.05*), as analyzed by one-way ANOVA followed by Bonferroni's post-hoc test. (*E*) complex-III protein expression was determined by immunobloting using pure mitochondrial protein isolated from control and 150 µM capsaicin treated BxPC-3 cells for indicated time periods or (*F*) Pretreatment with catalase (2000 u/ml) or EUK-134 (50 µM) for 1 h followed by 150 µM capsaicin for 24 h. Expression of complex-III protein was determined by immunoblotting from isolated pure mitochondria as described in the method. Each blot was stripped and reprobed with anti-Cox-IV antibody to ensure equal protein loading. These experiments were performed three times independently with similar result obtained in each experiment.

Mitochondrial ETC complexes are the major generators of ROS in cells and tissues. Since we observed ROS generation by capsaicin, we wanted to see if mitochondria are involved in this process. We therefore determined the enzymatic activities and expression of mitochondrial complex-I, complex-II, complex-III and complex-IV in capsaicin treated BxPC-3, AsPC-1, HPDE-6 and BxPC-3 ρ^0^ cells. Capsaicin treatment inhibits complex-I activity by about 5–20% in BxPC-3 and 2.5–9% in AsPC-1 cells respectively as compared to respective controls (Fig. 3A–B). On the other hand, as expected, capsaicin failed to inhibit complex-I activity in BxPC-3 ρ^0^ cells (which lack mitochondrial DNA) and normal HPDE-6 cells (Fig. 3C). Next, we wanted to investigate whether this decrease in complex-I activity can be attenuated by anti-oxidants. Our results reveal that pretreatment of cells with catalase or EUK-134 substantially blocked the decreases in complex-I activity by capsaicin (Fig. 3D). Further capsaicin treatment significantly decreased the protein levels of complex-I protein complex after 4 h of treatment in a time dependent study and catalase or EUK-134 prevented this change (Fig. 3E–F). Similarly, complex-III activity by capsaicin was inhibited by 8–75% in both BxPC-3 and AsPC-1 cells (Fig. 4A–B). Nonetheless, capsaicin failed to decrease complex-III activity in BxPC-3 ρ^0^ cells (Fig. 4C). A modest decrease in complex III activity was however observed in HPDE-6 cells by capsaicin treatment (Fig. 4C). The decrease in complex-III activity in BxPC-3 cells by capsaicin was attenuated by catalase and EUK-134 (Fig. 4D). In agreement with activity data, expression of complex-III protein complex was drastically reduced in BxPC-3 cells following capsaicin treatment (Fig. 4E). The effect of capsaicin on the protein level of complex-III was abrogated by catalase and EUK-134 (Fig. 4F). Our results show that mitochondrial complex-III is more involved in capsaicin mediated ROS generation as compared to complex-I. Capsaicin had no effect on complex-II and IV (data not shown). Taken together, these results indicate that inhibition of mitochondrial complex I and complex-III by capsaicin cause ROS generation.

### Effect of capsaicin on mitochondrial ATP generation

**Figure 5:**
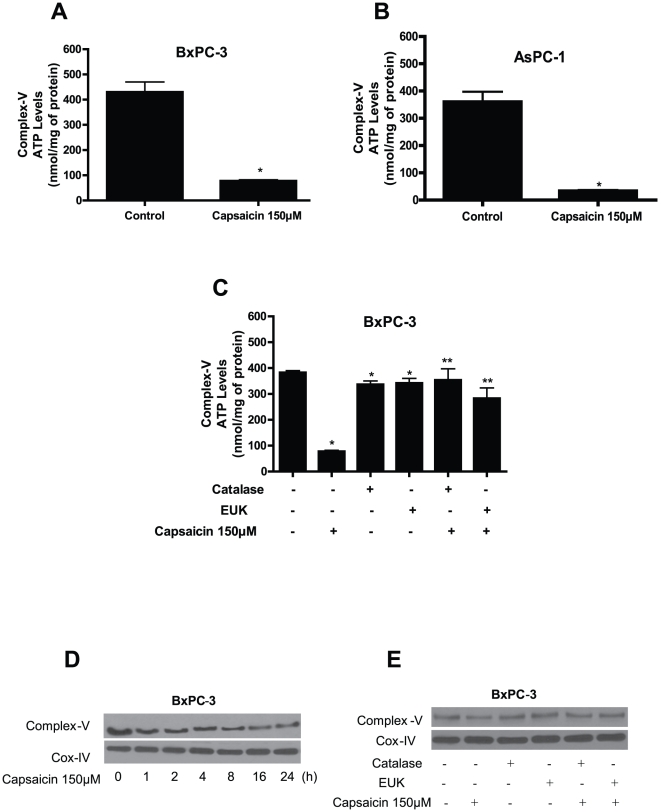
Effect of capsaicin on mitochondrial ATP-synthase (complex V) activity. (*A*) BxPC-3 (*B*) AsPC-1 cells were treated with DMSO or 150 µM capsaicin for 24 h, and (*C*) BxPC-3 cells were treated with catalase (2000 U/ml) or EUK-134 (50 µM) 1 h prior to 150 µM capsaicin treatment for 24 h and ATP-synthase activity was determined in pure mitochondria protein isolated from control and treated cells as described in the method section. Results are expressed as mean ± SD (n = 3) of three independent experiments. *Statistically significant when compared with control or ** statistically significant when compared with capsaicin treatment, as analyzed by one-way ANOVA followed by Bonferroni's post-hoc test (*P<0.05*). (*D*) Effect of capsaicin treatment on complex-V protein expression. BxPC-3 cells were treated with DMSO or 150 µM capsaicin for indicated time periods or (*E*) BxPC-3 cells were treated with catalase (2000 U/ml) or EUK-134 (50 µM) for 1 h prior to treatment with 150 µM capsaicin for 24 h. Expression of complex-V protein was determined by immunoblotting in the pure mitochondrial protein as described in the method section. Each blot was stripped and reprobed with anti-Cox-IV antibody to ensure equal protein loading. These experiments were performed three times independently with similar results obtained in each experiment.

Mitochondria are the major source of energy for the cells. We next wanted to know whether capsaicin mediated disruption of mitochondrial respiratory complexes affected ATP generation. To determine the levels of ATP, we evaluated complex-V ATP synthase activity in the mitochondria isolated from control and capsaicin treated BxPC-3 and AsPC-1 cells. The generation of ATP is through complex-V in the mitochondria. Capsaicin treatment depleted ATP levels by about 75% in both BxPC-3 and AsPC-1 cells as compared to control (Fig. 5A–B). We also observed that catalase and EUK-134 significantly prevented the decline in ATP levels in response to capsaicin treatment (Fig. 5C). To further confirm these observations, expression of mitochondrial complex-V protein was determined by western blotting. Our results reveal that capsaicin treatment decreased the expression of complex-V protein starting as early as 1 h but was more prominent at 16 and 24 h (Fig. 5D). This decline in complex-V expression was attenuated by catalase and EUK-134 (Fig. 5E). Overall, our results demonstrate that capsaicin treatment drastically disrupts mitochondrial functions pushing the cells towards apoptosis.

### Capsaicin disrupts mitochondrial membrane potential and cause oxidation of mitochondrial lipid

**Figure 6:**
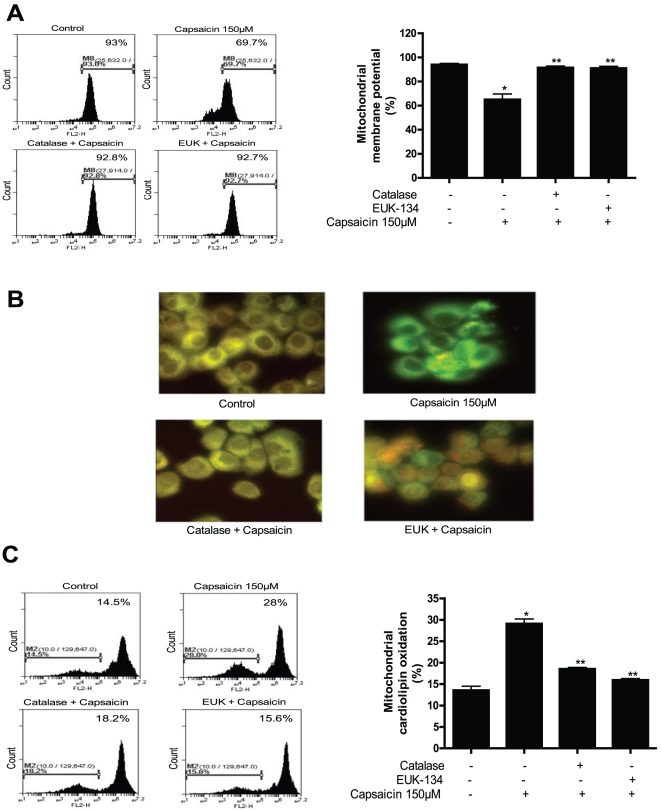
Capsaicin disrupts mitochondrial membrane potential and cause oxidation of mitochondrial lipid. (*A*) BxPC-3 cells were treated with catalase (2000 U/ml) or EUK-134 (50 µM) for 1 h followed by 150 µM capsaicin for 24 h and the change in mitochondrial membrane potential was determined by staining the cell with mitochondrial membrane sensitive dye TMRM and analyzed by flow cytometry. Right panel shows quantitation of mitochondrial membrane potential. (*B*) Effect of capsaicin on mitochondrial lipid peroxidation. BxPC-3 cells were treated with catalase (2000 U/ml) or EUK-134 (50 µM) for 1 h prior to treatment with 150 µM capsaicin for 24 h and stained with nonyl acridine orange (NAO) to detect oxidation of cardiolipin, a mitochondrial membrane lipid component by fluorescence microscopy, and (*D*) flow cytometry and right panel shows quantitation of mitochondrial cardiolipid oxidation. Representative result from three experiments performed independently. *Statistically different when compared with control (*P<0.05*) or **statistically different when compared with capsaicin treatment alone (*P<0.05*), as analyzed by one-way ANOVA followed by Bonferroni's post-hoc test.

Excessive intracellular ROS lead the cells to apoptosis by disrupting mitochondrial membrane potential. The change in mitochondrial membrane potential by capsaicin was thus determined by staining the cell with mitochondrial membrane sensitive dye TMRM and analyzed by flow cytometry. We found that capsaicin treatment significantly decreased the mitochondrial membrane potential in BxPC-3 cells by 26% as compared to control (Fig. 6A). To confirm whether capsaicin mediated ROS causes change in mitochondrial membrane potential, catalase and EUK-134 were used. Pretreatment of cells with both antioxidants followed by capsaicin completely prevented the drop in mitochondrial membrane potential (Fig. 6A). We further examined the possibility whether capsaicin preferentially induce mitochondrial lipid peroxidation in BxPC-3 cells. For this purpose, cells were stained with nonyl acridine orange (NAO) to detect oxidation of cardiolipin, a mitochondrial membrane lipid component, by fluorescence microscopy and flow cytometry [37]. Cardiolipin is exclusively present in mitochondria and after being labeled with NAO and exhibits yellow fluorescence. When we analyzed our cells under the fluorescent microscope, we observed that almost all the cells from control group were exhibiting yellow color. However, the yellow staining decreased and turned into green in capsaicin treated cells indicating drastic oxidation of cardiolipin (Fig. 6B). Nonetheless, catalase and EUK-134 completely prevented the oxidation of cardiolipin (Fig. 6B). These results were confirmed by flow cytometry where we observed that capsaicin causes cardiolipin oxidation in BxPC-3 cells as shown by a shift of NAO fluorescence towards left (Fig. 6C). We further used catalase and EUK-134 to see whether the oxidation of cardiolipin can be prevented. We found that addition of catalase or EUK-134 almost completely blocked the shift of NAO staining (Fig. 6C) suggesting that the decrease of NAO fluorescence was due to oxidation of mitochondrial lipid cardiolipin by mitochondrial ROS.

### Capsaicin-induced apoptosis is attenuable by anti-oxidants

**Figure 7:**
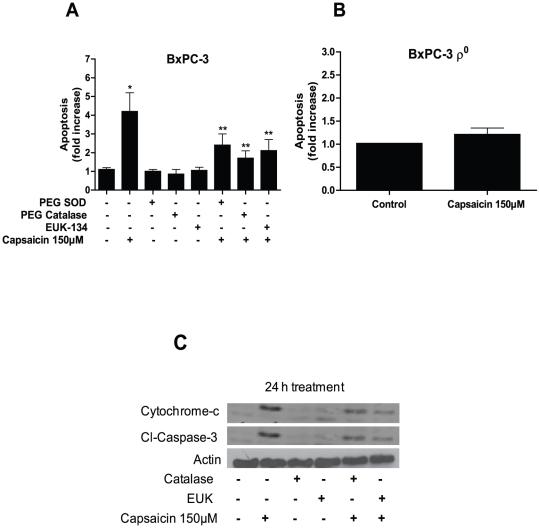
Anti-oxidants prevent capsaicin-induced apoptosis. (*A*) BxPC-3 cells were pretreated with PEG-SOD (100 U/ml), PEG-catalase (500 U/ml) or EUK-134 (50 µM) for 1 h and then treated with DMSO or 150 µM capsaicin for 24 h, (*B*) BxPC-3 ρ^0^ cells were treated with DMSO or 150 µM capsaicin for 24 h, and apoptosis was determined using annexin-V/FITC and propidium iodide and analyzed by flow cytometery. Results are expressed as mean ± SD (n = 3) of three independent experiments. *Statistically different when compared with control (*P<0.05*) or ** statistically significant when compared with capsaicin treatment (*P<0.05*), as analyzed by one-way ANOVA followed by Bonferroni's post-hoc test. (C) Cytochrome-c and Cl-caspase-3 were determined by immunoblotting in BxPC-3 cells pretreated with catalase (2000 U/ml) or EUK-134 (50 µM) for 1 h prior to treatment with 150 µM capsaicin for 24 h. Each blot was stripped and reprobed with anti-actin antibody to ensure equal protein loading. These experiments were performed three times independently and similar results were obtained.

We observed that capsaicin causes ROS generation by disrupting mitochondrial function. Once mitochondrial functions are disrupted, cytochrome-c is released from the mitochondria into the cytosol and activate caspase-3 cascade leading the cells into apoptosis. We wondered whether catalase and EUK-134 could abrogate capsaicin induced apoptosis. As shown in Fig. 7A, PEG-SOD, PEG-catalase and EUK-134 significantly protected BxPC-3 cells from capsaicin induced apoptosis. These results were further confirmed by evaluating the release of cytochrome-c and cleavage of caspase-3 by western blotting. Our results reveal that both catalase and EUK-134 significantly prevented the release of cytochrome-c into the cytosol and cleavage of caspase-3 mediated by capsaicin (Fig. 7C). It is noteworthy that BxPC-3 ρ^0^ cells, which are unable to produce ROS through mitochondria, were totally resistant to the apoptosis inducing effects of capsaicin (Fig. 7B), confirming the involvement of mitochondria in capsaicin mediated ROS generation and apoptosis.

### Capsaicin treatment disrupts cellular redox homeostasis resulting in oxidative stress

**Figure 8:**
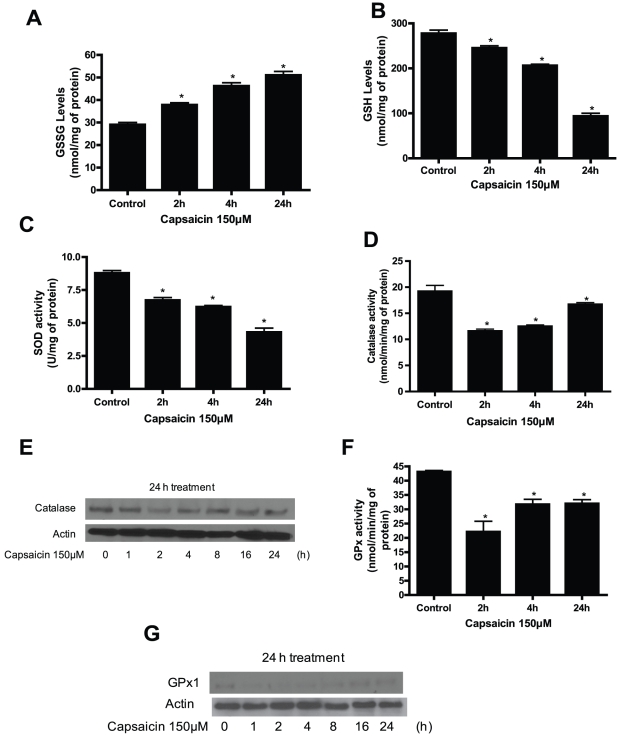
Capsaicin treatment disrupts cellular redox homeostasis resulting in oxidative stress. (A) Effect of capsaicin on the levels of oxidized glutathione (GSSG). BxPC-3 cells were treated with DMSO or 150 µM capsaicin for 2, 4 and 24 h and GSSG and (*B*) GSH levels were determined using a commercially available kit. These experiments were repeated twice with similar results obtained each time. (*C*) SOD, (*D*) Catalase, (*F*) GPx activities were determined as described in the method section. BxPC-3 cells were treated with DMSO or 150 µM capsaicin for 2, 4 and 24 h. Results are expressed as mean ± SD (n = 3) of three independent experiments. *Statistically different when compared with control (*P<0.05*) as analyzed by one-way ANOVA followed by Bonferroni's post-hoc test. *(E) and (G)* Expression of catalase and GPx1 were determined by immunoblotting of BxPC-3 cells treated with DMSO or 150 µM capsaicin for indicated time period. Each blot was stripped and reprobed with anti-actin antibody to ensure equal protein loading. These experiments were performed three times independently and similar results were obtained.

Redox homeostasis in a cell is due to a fine balance between the intracellular ROS and ROS scavenging antioxidants and enzyme systems. Reduced GSH is an intracellular antioxidant and is known to maintain cellular redox balance. We therefore measured intracellular GSH levels and also determined the levels of oxidized form of GSH (GSSG). As shown in Fig. 8A, capsaicin treatment significantly increased GSSG levels; and decreased GSH levels indicating the shift of redox equilibrium towards pro-oxidant state (Fig. 8A–B). The other enzyme systems which play role in redox balance include superoxide dismutase (SOD), catalase and glutathione peroxidase (GPx). Superoxide radicals are generated by complex-I and complex-III of the mitochondria and are rapidly converted into hydrogen peroxide due to dismutation by superoxide dismutase. As shown in Fig. 8C, capsaicin treatment inhibited 23–51% SOD activity in a time-dependent study. The other two enzymes (catalase and GPx) are involved in detoxifying intracellular peroxides including hydrogen peroxide. Our results demonstrate that capsaicin significantly reduced the enzymatic activity of catalase within 2 h of treatment (Fig. 8D). These observations were confirmed by catalase protein expression. We observed that catalase expression was decreased after 2 h of capsaicin treatment (Fig. 8 D–E). Throughout our studies, we observed that catalase or EUK-134 supplementation prevented ROS generation and protected the cells from the deleterious effects of capsaicin, clearly indicating that catalase plays a critical role in capsaicin mediated oxidative stress and apoptosis in pancreatic cancer cells. Since glutathione peroxidase is another important enzyme that utilizes GSH as a substrate to detoxify hydrogen peroxide, we determined its enzymatic activity and protein expression. As can be seen in Fig. 8 F–G, capsaicin reduced GPx activity and expression in BxPC-3 cells in response to capsaicin treatment. In fact, the expression of GPx was significantly reduced just after 1 h of capsaicin treatment. Taken together, our results suggest that depletion of GSH level and inhibition of SOD, catalase and GPx by capsaicin disturbs the cellular redox homeostasis resulting in increased oxidative stress.

### Ectopic expression of catalase protect the cells from capsaicin mediated ROS generation and apoptosis

**Figure 9:**
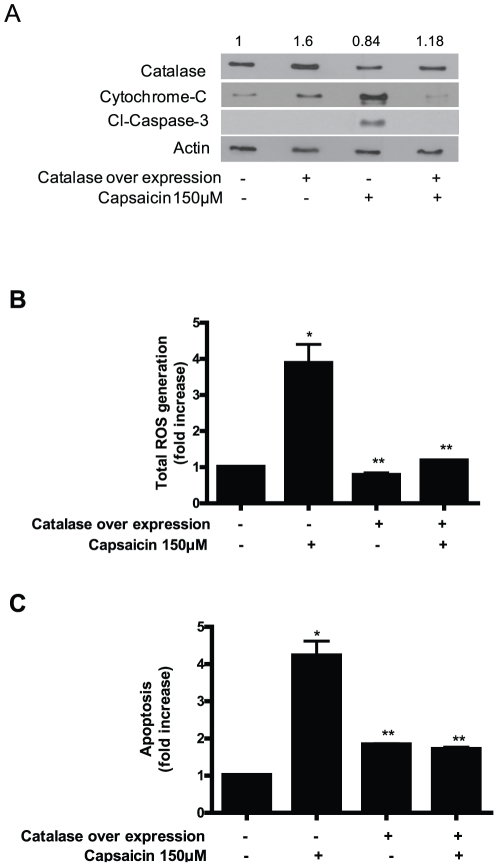
Catalase overexpression protect the cells from capsaicin mediated ROS generation and apoptosis. (*A*) BxPC-3 cells were transiently transfected with catalase expressing plasmid for 24 h followed by treatment with DMSO or 150 µM capsaicin for another 24 h and the expression of catalase, cytochrome-c and Cl-caspase-3 were evaluated by immunoblotting. Each blot was stripped and reprobed with anti-actin antibody to ensure equal protein loading. These experiments were performed two times independently with similar observations made in each experiment. (*B*) ROS and (*C*) apoptosis assay were determined in catalase tranfected BxPC-3 cells followed by treatment with or without 150 µM capsaicin for 24 h. Results are expressed as mean ± SD (n = 3) of three independent experiments. *Statistically different when compared with control (*P<0.05*) or **statistically different when compared with capsaicin treatment alone (*P<0.05*), as analyzed by one-way ANOVA followed by Bonferroni's post-hoc test.

Since we observed that capsaicin mediated ROS generation, mitochondrial damage and apoptosis were attenuated by catalase or EUK-134, we next wanted to see if ectopic expression of catalase can protect the cells from capsaicin mediated damage. We transiently transfected the cells with catalase expressing plasmid and were able to achieve about 1.6 fold overexpression of catalase as compared to vector control (Fig. 9A). The decrease in catalase expression by capsaicin treatment was blocked in the cells transfected with catalase (Fig. 9A). Further, catalase over expressing BxPC-3 cells completely blocked total ROS generation by capsaicin and protected the cells from apoptosis as compared to capsaicin treated vector transfected cells (Fig. 9B–C). The release of cytochrome c and cleavage of caspase-3 was also completely blocked in the cells over expressing catalase (Fig. 9A). These results clearly establish the protective role and involvement of catalase in capsaicin mediated mitochondrial damage and cell death.

### Capsaicin treatment reduces antioxidant levels in pancreatic tumor xenografts in vivo

**Figure 10:**
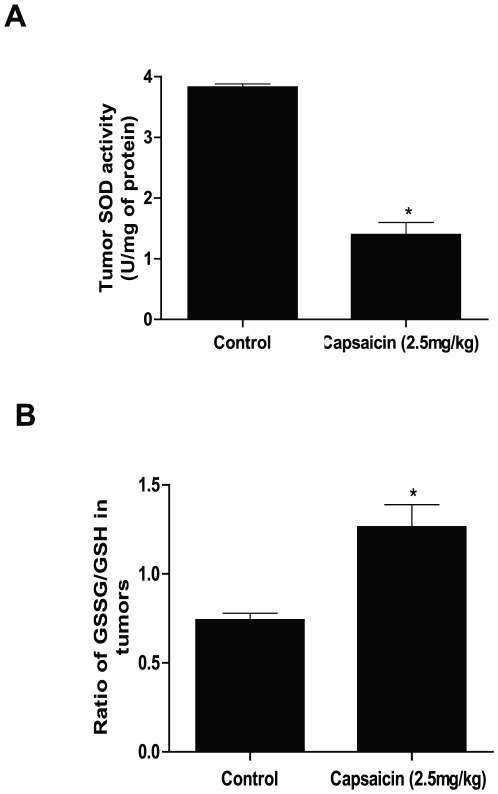
Capsaicin treatment generates oxidative stress *in vivo* in pancreatic tumors by reducing antioxidants. Tumors from control and 2.5 mg/kg (5days/week for 6 weeks) capsaicin treated mice were analyzed for (*A*) SOD enzymatic activity and (*B*) GSSG and GSH levels. Results are expressed as mean ± SD (n = 3) of three independent tumors. *Statistically different when compared with control (*P<0.05*).

In our previously published studies, we have shown that treatment of athymic nude mice with 2.5 mg/kg capsaicin 5 days a week by oral gavage for six weeks significantly suppressed the growth of AsPC-1 tumor xenografts [34]. To establish whether antioxidant levels in the tumors were associated with capsaicin-mediated tumor growth suppression, the tumors from control and capsaicin treated mice were used to evaluate SOD enzymatic activity and the levels of GSH and GSSG. The SOD activity in the tumors of capsaicin treated mice was reduced by 60% as compared to control tumors (Fig. 10A). Consistent with our cellular results, we observed about 1.8 fold increase in GSSG/GSH level in capsaicin treated tumors as compared to control tumors indicating oxidative stress (Fig. 10B). Taken together, our results suggest that decreased antioxidants and increased pro-oxidants may be associated with capsaicin-mediated tumor growth suppression *in vivo*.

## Discussion

Mitochondria are a major physiological source of ROS, which are generated due to incomplete reduction of oxygen during normal mitochondrial respiration. Excessive ROS that are generated under certain pathological conditions acts as mediator of apoptotic signaling pathway. Under normal physiological conditions, mitochondria contain sufficient levels of antioxidants that prevent ROS generation and oxidative damage. However, under circumstances in which excessive mitochondrial ROS are produced or when antioxidant levels are depleted, oxidative damage to mitochondria occurs. Our current results shows that capsaicin induced apoptosis in BxPC-3 and AsPC-1 cells but not in HPDE-6 cells was associated with ROS generation. The ROS generation by capsaicin was due to marked inhibition of mitochondrial electron transport chain (ETC) complexes-I and III and downregulation of antioxidants such as GSH, catalase, SOD and GPx indicating the involvement of mitochondria. On the other hand, ρ^0^ cells derived from BxPC-3 cells, which lack normal oxidative phosphorylation were unable to cause ROS generation and were totally resistant to the apoptosis inducing effects of capsaicin.

Mitochondrial ETC has been recognized as the major intracellular source of reactive oxygen species [38]. Complex-I and complex-III of ETC are the major sites for ROS generation. The present study provides convincing experimental data to prove that ROS generation by capsaicin in pancreatic cancer cells is through ETC complex-I and complex-III and not through complex-II and IV. Capsaicin (150 µM, 24 h) treatment cause significant decrease in ETC complex-I and complex-III activities in BxPC-3 and AsPC-1 cells but not in normal HPDE-6 cells. To confirm whether capsaicin mediated ROS were mitochondria derived; we generated BxPC-3 ρ^0^ cells in which mitochondrial DNA is depleted. The BxPC-3 ρ^0^ cells were completely resistant to ROS generation and apoptosis induced by capsaicin as compared with wild type BxPC-3 cells suggesting that a functional electron transport chain is required for capsaicin mediated ROS generation. These results are consistent with previous studies where ETC complexes were involved in ROS generation [38]. However, in contrast to those studies where whole cell lysate was used to determine ETC complex activities [38], we used pure mitochondria from control and capsaicin treated cells to evaluate ETC activity. ROS once generated cause oxidation of critical redox sensitive proteins and lipids leading to mitochondrial damage. Our results clearly show that capsaicin treatment, cause massive oxidation of cardiolipin, which is specifically present in the mitochondria. Mitochondrial damage due to oxidation of cardiolipin has been documented in a recent study [37]. Cytochrome c preferentially binds to cardiolipin and is liberated upon oxidation of cardiolipin [39]. In agreement, our results show the release of cytochrome c into cytosol by capsaicin treatment, which could be due to cardiolipin oxidation. Our results also demonstrate massive depletion of ATP as evaluated by complex-V ATP synthase activity. ETC complex forms a transmembrane potential (Δψ). ATP synthase uses potential energy stored in Δψ to phosphorylate ADP. However, under certain pathological conditions, the Δψ can collapse resulting in the release of molecules from the mitochondria into the cytosol [34]. Our result do show decrease in Δψ and release of cytochrome c into the cytosol in response to capsaicin treatment. Further, ATP production was shown to be highly sensitive to complex-III inhibition in a previous report [40]. In agreement, our results also show a relationship between complex III inhibition and ATP depletion.

Cellular redox homeostasis is maintained by a fine balance between antioxidants and pro-oxidants. Glutathione is a critical intracellular antioxidant responsible for maintaining redox balance. GSH can be oxidized to form GSSG and the ratio of GSH/GSSG is an indicator of oxidative stress in the cells [37]. High concentrations of GSSG can oxidatively damage many critical enzymes. Our results reveal that capsaicin treatment caused time dependent increase in the levels of GSSG and decrease in GSH levels in BxPC-3 cells. Similar observations were made in the tumors of capsaicin treated mice as compared to the tumors from control mice. The GSSG levels increased and GSH level decreased hence the ratio of GSSG/GSH increased in the tumors of capsaicin treated mice. Superoxide dismutase (SOD) is an enzyme responsible for dismutating superoxide radicals, which are generated in the mitochondria by ETC complex I and complex III. Over-expression of SOD has been shown in lung tumors as compared to normal tissues suggesting its role in lung carcinogenesis [41]. Moreover, SOD was recently identified as a target for the selective killing of cancer cells [42]. Our results clearly show that capsaicin treatment significantly decreased SOD activity in BxPC-3 cells and AsPC-1 tumor xenografts. Glutathione peroxidase (GPx) is an important enzyme that utilizes GSH as a substrate to detoxify intracellular peroxides including hydrogen peroxide. Capsaicin treatment resulted in the significant inhibition of GPx activity and expression in BxPC-3 cells. These results indicate that capsaicin deplete GSH level and inhibit GSH dependent anti-oxidant enzymes resulting in the accumulation of ROS in pancreatic cancer cells leading to mitochondrial damage. In addition catalase is another important enzyme which is responsible for detoxifying hydrogen peroxide to water. Consistently, we observed that PEG-SOD, PEG-catalase, catalase or EUK-134 (a cell permeable catalase mimetic) prevented capsaicin mediated ROS generation by complex-I and complex-III, ATP depletion, mitochondrial damage and apoptosis, indicating the involvement of catalase. As a proof-of-concept, over-expression of catalase by transient transfection completely blocked capsaicin mediated ROS generation and apoptosis in BxPC-3 cells demonstrating its critical role in the survival of pancreatic cancer cells.

Most of the cancer cells have higher levels of ROS that helps in proliferation and cell growth [37]. Due to elevated ROS, cancer cells are highly dependent on their antioxidant system to maintain redox balance and hence are more susceptible to further oxidative stress. In contrast, normal cells are more resistant to oxidative stress due to the fact that these cells have lower levels of ROS and increased levels of antioxidants. Hence any agent that increases intracellular ROS in cancer cells may increase ROS to a toxic level resulting in mitochondrial damage and cell death as shown in our model. It is noteworthy that several agents such as Elesclomol or Trisenx are currently being used for the treatment of metastatic melanoma and acute promyelocytic leukemia respectively [43]. Both of these agents selectively kill cancer cells by increasing ROS generation [43].

We and others have shown previously that administration of 2.5 or 5 mg/kg capsaicin orally or subcutaneously suppress pancreatic and prostate tumor xenografts *in vivo* respectively [34], [44] . In the present study, 2.5 mg/kg capsaicin was given to mice by oral gavage, which is 0.202 mg/kg when converted to human equivalent dose (HED) and equates to 13.2 mg dose of capsaicin for a 60 kg person [45]. However, further pharmacokinetic, bioavailability and clinical studies are needed to validate these correlations.

Taken together our studies provide detailed mechanism how capsaicin treatment causes ROS generation through mitochondria and depleted intracellular antioxidants resulting in mitochondrial damage and apoptosis in pancreatic cancer cells. On the other hand, normal pancreatic epithelial cells were resistant to the effects of capsaicin.

## Materials and Methods

### Chemicals and Antibodies

Capsaicin (purity>99%), propidium iodide, anti-actin, H_2_O_2_, PEG-SOD, PEG-catalase, catalase, EUK-134, NADH-dipotassium, BSA-FFA, rotenone, KCN, oligomycin, ATP-magnesium, albumin and cytochrome-c were obtained from Sigma (St. Louis, MO). The antibodies against cytochrome c, Cl-caspase-3, Cl-caspase-9, Cl-PARP, GPx1, CoxIV were purchased from Cell Signaling (Danvers, MA) and complex-I, complex-III and complex-V were purchased from Mito Sciences Inc. (Eugene, OR). Mitochondria isolation kit for mammalian cells and enhanced chemiluminescence kit were procured from Thermo Scientific (Pierce, Rockford, IL). The antibodies against catalase were purchased from Calbiochem. The specific probes HE, DCFDA, TMRM, Goat anti-mouse IgG (H+L) were obtained from Molecular Probes (Eugene, OR). Apoptosis detection kit Annexin V-FITC was procured from (BD Bio-Sciences, Inc. La Jolla, CA). Catalase, superoxide dismutase (combined Cu/Zn, Mn, and Fe-SOD) and glutathione peroxidase assay kits were purchased from Cayman Chemical (MI, USA).

### Cell Culture

Human pancreatic cancer cell lines BxPC-3 and AsPC-1 were procured from American Type Culture Collection (Rockville, MD). Monolayer cultures of AsPC-1 and BxPC-3 cells were maintained in RPMI medium supplemented with 10% fetal bovine serum, PSN antibiotic mixture (10 ml/L) (Gibco BRL, Grand Island, NY), 2 mM L-glutamine, 10 mM HEPES, 1 mM sodium pyruvate and 20% glucose. Normal HPDE-6 cells from human pancreas were provided by Dr. Ming-Sound Tsao, and cultured in keratinocyte serum-free medium supplemented with 4 mM L-glutamine and PSN antibiotic mixture (10 ml/L).

### Generation of BxPC-3 derived ρ^0^ cells

BxPC-3 derived ρ^0^ cells were generated and maintained by incubation of BxPC-3 with 60 ng/ml of ethidium bromide and 50 mg/ml of uridine for 12 weeks as described by King and Attadi et al [36]. Absence of mtDNA in ρ^0^ clones of BxPC-3 was confirmed by PCR as reported by Hail et al [35]. All the cultures were maintained at 37°C in a humidified chamber of 95% air and 5% CO_2_.

### Generation of reactive oxygen species (ROS)

Intracellular ROS generation was determined by measuring the levels of super-oxide and hydrogen peroxide produced in the cells by flow cytometry after staining the cells with hydroethidine and 6-carboxy-2, 7-dichlorodihydrofluorescein diacetate (DCFDA) as described by us previously [46], [47]. DCFDA is cell permeable and is cleaved by nonspecific esterases and oxidized by intracellular peroxides to form fluorescent 2, 7-dichlorofluorescin (DCF). Briefly, 0.3×10^6^ cells were plated in each well of six well plates and allowed to attach overnight and exposed to either DMSO or 150 µM capsaicin for varying time periods. Cells were further incubated with 2 µM hydroethidine and 5 µM DCFDA at 37°C for 25 min. Subsequently, cells were removed, washed and resuspended in PBS and analyzed for ROS generation using Accuri C6 flow cytometer. Approximately 10,000 cells were evaluated for each sample. In all determinations, cell debris and clumps were excluded from the analysis. In another experiment, cells were pretreated for 1 h with PEG-SOD (100 U/ml), PEG-catalase (500 U/ml), catalase (2000 U/ml) or EUK-134 (50 µM) prior to capsaicin treatment and analyzed of ROS generation. The results from catalase and PEG-catalase in terms of blocking ROS were very similar; hence both the antioxidants were used in the present study. We did not observe any toxicity to the cells with either of the antioxidants.

### Determination of apoptosis

Apoptosis inducing effects of capsaicin in BxPC-3, AsPC-1, HPDE-6 and BxPC-3 ρ^0^ cells was determined by flow cytometery using annexin-V/FITC and propidium iodide as described by us previously [47]. About 0.3×10^6^ cells were plated in each well of 6-well plate and treated with varying concentrations of capsaicin for 24 h or treated with 150 µM capsaicin for 2, 4, and 24 h. Apoptosis was determined using APOPTEST™-FITC kit according to manufacturer's instructions and analyzed by Accuri C6 flow cytometer. In another experiment, cells were treated for 1 h with PEG-SOD (100 U/ml), PEG-catalase (500 U/ml) or EUK-134 (50 µM) or prior to treatment with 150 µM capsaicin for 24 h and analyzed for apoptosis.

### Determination of oxidative damage to mitochondrial membrane and membrane potential

Mitochondrial membrane lipid peroxidation was detected by measuring the oxidation of intracellular cardiolipin, using 10-*N*-nonyl Acridine Orange (NAO) (Molecular Probes), a probe specific for mitochondrial membrane cardiolipin [37]. Briefly, BxPC-3 cells were incubated for 24 h with DMSO or 150 µM capsaicin, washed and then incubated for 25 minutes at room temperature with 5 µM NAO. After being washed with PBS, the cells were observed under the fluorescence microscope with FITC filter or by flow cytometry. Mitochondrial membrane potential was analyzed by flow cytometry using the membrane potential sensitive dye TMRM, which is taken up by the mitochondria of the normal cells in a potential dependent manner. TMRM changes the intensity but not the emission spectra in response to membrane potential and the signal was analysed in FL2 channel, equipped with band pass filter 580±30 nm. Briefly, control and capsaicin treated cells were incubated with 50 nM TMRM at 37°C for 20 min. Cells were then harvested, washed and resuspended in cold PBS. Approximately 10,000 cells were evaluated for each sample and forward scatter versus side scatter was used to gate the viable population of cells. In all determinations, cell debris and clumps were excluded from the analysis.

### Mitochondrial electron transport chain (ETC) complex activities

The integrated enzymatic activities of mitochondrial complex-I and complex-III were determined in the pure mitochondria isolated from control and 150 µM capsaicin treated BxPC-3, AsPC-1, HPDE-6 and BxPC-3 ρ^0^ cells using mitochondria isolation kit (Pierce, Rockford, IL). Mitochondrial complex-I activity was measured by determining the decrease in NADH absorbance at 340 nm that leads to the reduction of ubiquinone (CoQ1) to ubiquinol as described by Ragan *et al.* with slight modification [48]. Briefly cells were plated at a density of 5×10^6^ in 150-mm culture dishes and allowed to attach overnight and then treated with DMSO or 150 µM capsaicin for 2, 4 and 24 h. Cells were collected by scraping, washed with PBS and pure mitochondria were isolated using the above mentioned mitochondria isolation kit according to the manufacture instruction. The assay was initiated by the addition of 50 µM CoQ1 to the reaction mixture containing 20 µg of pure mitochondrial protein, 20 mM of potassium phosphate buffer (pH 7.2), 10 mM MgCl_2_, 0.15 mM NADH, 2.5 mg BSA-FFA and 1 mM KCN. After monitoring the reaction for 5 min at 30°C, 10 µM rotenone was added and the reaction was monitored for an additional 5 min. The activity was calculated using the extinction co-efficient of 6.22 mM^−1^ cm^−1^ for NADH and expressed as nmol/min/mg protein.

Mitochondrial complex-III (ubiquinol cytochrome c reductase) activity was measured by monitoring the reduction of cytochrome-c by ubiquinol at 550 nm as described by Ragan *et al* [48]. The enzymatic reaction is of first order which depend on the concentration of both UQ_2_H_2_ and cytochrome-c. The reaction mixture contained 35 mM potassium phosphate buffer pH 7.2, 1 mM EDTA, 5 mM MgCl_2_, 1 mM KCN, 5 mM rotenone, 15 mM cytochrome c and the 20 µg of mitochondrial protein and was initiated by the addition of 15 µM ubiquinol. The activity of complex-III was calculated by the pseudo-first order constant *K* and the results were represented as *K*/min/mg protein. Mitochondrial complex-II and IV were also determined according to Hatefi and Stiggal [49] and Wharton and Tzagoloff [50].

### Mitochondrial ATP-synthase (complex V) assay

Mitochondrial ATP-synthase assay was determined at 660 nm, according to the method described by Taussky and Shorr [51]. Pure mitochondrial protein from control and 150 µM capsaicin treated BxPC-3 and AsPC-1 cells were incubated in 1 ml of Na^+^ medium at 30°C for 10 min in the presence or absence of oligomycin (1 µg/mg of mitochondrial protein). The reaction was started by the addition of 1 mM ATPMg^2+^ at pH 7.4, and the samples were further incubated for 15 min at 30°C. The reaction was stopped by the addition of ice cold 40% TCA and protein was pelleted by centrifugation at 3,000×*g*, for 5 min. The absorbance in the supernatant was measured at 660 nm, 5 min after the addition of molibdate reagent and the amount of *P*i produced was determined using a phosphate standard curve. The ATP-synthase activity was determined by difference between the activity obtained in the presence or in the absence of oligomycin. Results were expressed as nmol *P*i/mg protein.

### Determination of Glutathione (GSH) and GSSG levels

Glutathione level was determined in BxPC-3 cells by glutathione kit obtained from Cayman Chemical (Ann Arbor, MI) as described by us previously [34]. Briefly cells were plated at a density of 1×10^6^ in 100-mm culture dishes and allowed to attach overnight and then treated with DMSO or 150 µM capsaicin for 2, 4 and 24 h. Cells were collected by scraping, washed with PBS, and cell lysate was used for determination of GSH level using the above mentioned kit according to the manufacture's instruction. To determine GSSG levels, GSH was masked by 2-vinyl pyredine for 1 h before the assay. The samples were read at 405 nm at 5 min intervals for 30 min. The GSH and GSSG were evaluated by comparison with standards and normalized with protein content.

In our previous studies, we have shown that oral feeding of 2.5 mg/kg capsaicin 5days/week for six weeks significantly suppressed the growth of AsPC-1 tumor xenografts in athymic nude mice [34]. In the present studies, tumors from control and capsaicin treated mice were homogenized and the GSH and GSSG levels were estimated as described above.

### Determination of Catalase, Superoxide Dismutase (SOD) and Glutathione Peroxidase (GPx) activities

The activities of catalase, SOD and glutathione peroxidase were determined in the mitochondria obtained from control and capsaicin treated BxPC-3 cells using catalase, SOD and GPx assay kit obtained from Cayman Chemical (Ann Arbor, MI). Catalase activity is based on the reaction of the enzyme with methanol in the presence of an optimal concentration of H_2_O_2_. The formaldehyde produced is measured colorimetrically at 540 nm with 4-amino-3-hydrozino-5-mercapto-1, 2, 4-triazole as the chromogen which upon oxidation changes from colorless to a purple color. SOD activity kit utilizes tetrazolium salt for detection of superoxide radicals generated by xanthine oxidase and hypoxanthine. One unit of SOD is defined as the amount of enzyme needed to exhibit 50% dismutation of the superoxide radical. The SOD assay kit measures combined activity of Cu/Zn, Mn, and Fe-SOD. GPx activity measures the rate of NADPH oxidation to NADP^+^, which is accompanied by a decrease in absorbance at 340 nm. One GPx unit is directly proportional to the amount of NADPH consumed in nmol per minute at 23–25°C and pH 7.6. Briefly, BxPC-3 cells were plated at a density of 5×10^6^ in 150-mm culture dishes and allowed to attach overnight and then treated with DMSO or 150 µM capsaicin for 2, 4 and 24 h. Cells were collected by scraping, washed with PBS, and pure mitochondria was isolated using the above mentioned mitochondrial isolation kit according to the manufacture's instruction. The tumors from control and capsaicin (2.5 mg/kg, 5days/week) treated mice [34] were homogenized in the buffer provided in the SOD assay kit and the activity was determined according to the manufacturer's instructions.

### Catalase transfection

pZEO SV2 mitochondrial catalase and empty vector pZEO SV2+ were kindly provided by Dr. Erin Moore (Albany Medical College, New York). About 0.3×10^6^ cells were plated in each well of 6-well plate and allowed to attach overnight. After cells were washed with OPTI-MEM serum free medium (Invitrogen), 1 µg of mitochondrial DNA was transfected in BxPC-3 cells using Lipofectamine™ 2000 reagent according to the manufactures protocol (Invitrogen) and as described by us previously [52]. After 6 h of incubation, medium was exchanged to complete RPMI medium containing 10% serum and antibiotics. After 24 h incubation cells were treated 0.1% DMSO or with 150 µM capsaicin for 24 h. Cells lysates were prepared and 10 µg of protein was analysed by western blotting. ROS generation and apoptosis assays were also performed in catalase transfected cells.

### Western blot analysis

Cells were exposed to various concentrations of capsaicin for 24 h or 150 µM capsaicin for 0, 1, 2, 4, 8, 16, 24 h and lysed on ice as described by us previously [53] . In a separate experiment cells were pre-treated with catalase (2000 U/ml) and EUK (50 µM) for 1 h followed by treatment with 150 µM capsaicin for 24 h. Whole-cell extracts were prepared by washing with cold PBS and lysed with above-mentioned lysis buffer. For cytochrome c determination, mitochondria free cytosol from control and capsaicin treated cells was prepared on ice in buffer containing 20 mM N-[(2-hydroxyethyl) piperazine-N-(2-ethanesulfonic acid)]– KOH (HEPES–KOH) pH 7.5, 10 mM KCl, 1.5 mM MgCl_2_, 1 mM EDTA–Na, 1 mM EGTA–Na, 1 mM dithiothreitol (DTT) containing 250 mM sucrose and mixture of protease inhibitors. The cell lysate was cleared by centrifugation at 14,000 g for 30 min. Cell lysate containing 10–80 µg protein was resolved by 6–12.5% sodium dodecyl sulfate-polyacrylamide gel electrophoresis (SDS-PAGE) and the proteins were transferred onto polyvinylidene fluoride membrane. After blocking with 5% non-fat dry milk in Tris buffered saline, membrane was incubated with the desired primary antibody overnight. Subsequently, the membrane was incubated with appropriate secondary antibody, and the antibody binding was detected by using enhanced chemiluminescence kit according to the manufacturer's instructions. Each membrane was stripped and re-probed with antibody against actin (1∶20000 dilutions) for cytosolic proteins and Cox IV (1∶1000) for mitochondrial proteins to ensure equal protein loading.

### Statistical analysis

All statistical calculations were performed using Graph Pad Prizm 5.0. Analysis of variance (ANOVA) was used to test the statistical significance of difference between control and treated groups followed by Bonferroni's post-hoc analysis for multiple comparisons. P-values less than 0.05 were considered statistically significant.

## Supporting Information

Figure S1 **Capsaicin produces H2O2 and superoxide separately in pancreatic cancer cells.** (A) and (B) BxPC-3 cells were treated with PEG-SOD (100 U/ml), PEG-catalase (500 U/ml) for 1 h followed by 150 µM capsaicin for 2 h and stained with HE and DCFDA and analysed for superoxide and hydrogen peroxide respectively. In Fig. (1C) BxPC-3 cells were treated with PEG-catalase (500 U/ml) for 1 h followed by 150 µM capsaicin for 2 h and stained HE and analysed for superoxide. *Statistically different when compared with control (P<0.05) or ** statistically different when compared with capsaicin treatment alone (P<0.05), as analyzed by one-way ANOVA followed by Bonferroni's post-hoc test.
(EPS)
